# Pathogenic Effect of *Prevotella intermedia* on a Mouse Pneumonia Model Due to Methicillin-Resistant *Staphylococcus aureus* With Up-Regulated α-Hemolysin Expression

**DOI:** 10.3389/fmicb.2020.587235

**Published:** 2020-10-07

**Authors:** Yu Yamashita, Kentaro Nagaoka, Hiroki Kimura, Masaru Suzuki, Tatsuya Fukumoto, Kasumi Hayasaka, Norihito Kaku, Yoshitomo Morinaga, Katsunori Yanagihara, Satoshi Konno

**Affiliations:** ^1^Department of Respiratory Medicine, Faculty of Medicine and Graduate School of Medicine, Hokkaido University, Sapporo, Japan; ^2^Division of Laboratory and Transfusion Medicine, Hokkaido University Hospital, Sapporo, Japan; ^3^Department of Laboratory Medicine, Nagasaki University Graduate School of Biomedical Sciences, Nagasaki, Japan

**Keywords:** methicillin-resistant *Staphylococcus aureus*, pneumonia, mouse model, *Prevotella intermedia*, quorum sensing system

## Abstract

**Background**: Methicillin-resistant *Staphylococcus aureus* (MRSA) is a common causative agent of pneumonia; however, the detailed mechanism underlying severe MRSA pneumonia, including association with oral hygiene or periodontitis, remains poorly characterized. In this study, we examined the pathogenic effect of *Prevotella intermedia*, a major periodontopathic pathogen, on MRSA pneumonia.

**Methods**: The pathogenic effect of the supernatant of *P. intermedia* (Pi Sup) was investigated in a murine MRSA pneumonia model, using several clinical strains; whereas the bactericidal activity of polymorphonuclear leukocytes (PMNs) was investigated *in vitro*. The effect of Pi Sup on messenger RNA (mRNA) expression of the toxin/quorum sensing system (rnaIII) was investigated by quantitative reverse transcription PCR both *in vitro* and *in vivo*.

**Results**: Mice infected by hospital-acquired MRSA (HA-MRSA) with Pi Sup exhibited a significantly lower survival rate, higher bacterial loads in the lungs, and higher α-hemolysin (hla) expression in the lungs, than those without Pi Sup. A similar effect of Pi Sup was not observed with MRSA strains producing Panton-Valentine leucocidin (PVL) or toxic shock syndrome toxin (TSST). *In vitro*, Pi Sup suppressed bactericidal activity of PMNs against the HA-MRSA strain. HA-MRSA was the clinical strain with the highest ability to proliferate in the lungs and was accompanied by time-dependent up-regulation of rnaIII and hla.

**Conclusions**: Our results provide novel evidence that the product of *P. intermedia* exerts a pathogenic effect on MRSA pneumonia, in particular with a strain exhibiting strong proliferation in the lower airway tract. Moreover, our results indicate that *P. intermedia* affects MRSA toxin expression *via* quorum sensing in a strain-dependent fashion, which might be important for understanding the pathogenesis of severe MRSA pneumonia.

## Introduction

Methicillin-resistant *Staphylococcus aureus* (MRSA) is an important pathogen in hospitals and intensive care units (Chastre et al., 2014). Although several countries have seen a decline in the prevalence of MRSA infections, possibly due to improved efforts in infection control (Navarro et al., 2008), the incidence of MRSA nosocomial pneumonia (MRSA-NP) still accounts for 16–40% of nosocomial pneumonia cases (Koulenti et al., 2009; Esperatti et al., 2010; Hanberger et al., 2011; Bouza et al., 2012; Chastre et al., 2014). A high (36–59%) in-hospital mortality rate has been reported among severe cases of MRSA-NP including ventilator-associated pneumonia (Hanberger et al., 2011; Bouza et al., 2012). In spite of its clinical importance, considerable gaps exist in our understanding of the mechanisms underlying severe MRSA-NP. Due to the difficulty in distinguishing between MRSA infection and colonization in sputum samples containing MRSA isolated from pneumonia cases, accurate diagnosis of MRSA-NP remains challenging and a new diagnostic approach is needed (Chastre et al., 2014; Nagaoka et al., 2014b). Risk factors for developing lethal MRSA-NP are not fully clarified, although several have been reported so far, such as prior use of antibiotics, history of chronic obstructive pulmonary disease, or high severity score on admission to the intensive care unit (Graffunder and Venezia, 2002; Dryden et al., 2010; Fukuta et al., 2012).

Poor oral hygiene has long been recognized as a potential risk factor for developing pneumonia, and oral care is widely recommended as a means of reducing MRSA incidence in a variety of situations, such as ventilator-associated pneumonia, pneumonia in elderly patients, and postoperative pneumonia associated with esophageal cancer (Yoneyama et al., 2002; Mori et al., 2006; Soutome et al., 2017). Oral care reduces bacterial colonization of the oropharynx, leading to fewer pathogenic bacteria in the oral cavity (Mori et al., 2006). However, descriptive data are not available, and a more detailed relationship between oral hygiene and pneumonia-causing MRSA needs to be established.

*Prevotella intermedia* is a gram-negative, rod-shaped, obligate anaerobe, as well as the major periodontopathic pathogen (Gharbia et al., 1994; Ximénez-Fyvie et al., 2000). The presence of *P. intermedia* has been reported frequently also in lower airway specimens, particularly in those of cystic fibrosis patients (Tunney et al., 2008; Worlitzsch et al., 2009; Nagaoka et al., 2017). As the pathogenic potential of *P. intermedia* in the respiratory tract, Ulrich demonstrated that extracellular toxins of *P. intermedia* are cytotoxic for human alveolar typeII cells and neutrophils (Ulrich et al., 2010). Similarly, we previously reported that the product of *P. intermedia* induced severe bacteremic pneumococcal pneumonia in mice with enhanced pneumococcal adhesion to lower airway cells (Nagaoka et al., 2014a), which suggested that periodontitis might be a potential risk factor for severe pneumococcal pneumonia. Recently, Takeshita conducted a clinical study using terminal restriction fragment length polymorphisms to survey the flora of the tongue coating in 343 elderly hospitalized or nursing home subjects (Takeshita et al., 2010). The study revealed that the tongue coating was characterized by a predominance of *Prevotella*, *Veillonella*, and *Treponema* species, and was closely associated with pneumonia development. Based on the above reports, we expected that investigating the relationship between *P. intermedia* and MRSA would help understand the effect of poor oral hygiene on MRSA pneumonia.

The present study aimed to determine whether *P. intermedia* exhibited a pathogenic effect on MRSA pneumonia in a murine model and its mechanism of interaction. We examined the effect of *P. intermedia* on MRSA virulence factors relevant for pneumonia pathogenesis, such as mRNA expression of protein A (spa) and α-hemolysin (hla; Parker and Prince, 2012). Given the emergence of community-acquired MRSA (CA-MRSA) pneumonia (Mediavilla et al., 2012), we also examined the effect of *P. intermedia* on expression of Panton-Valentine leucocidin (PVL) and toxic shock syndrome toxin (TSST), the two main toxins produced by CA-MRSA strains.

## Materials and Methods

### Bacterial Strains

The following MRSA clinical strains were used in this study: HUYM (saphylococcal cassette chromosome [SCC] mec type III; Yamashita et al., 2019), NU1643 (SCC mec type II; TSST+), and NU1516 (SCC mec type IV; PVL+). The HUYM strain was isolated at Hokkaido University School of Medicine; whereas the other two strains were isolated at Nagasaki University Hospital. SCC mec type and toxin gene of each strains were determined genetically using previously reported methods (Motoshima et al., 2010). *P. intermedia* strain PINU499 was obtained from and its supernatant (Pi Sup) was harvested as previously reported (Nagaoka et al., 2014a). Briefly, PINU499 was incubated using modified gifu anaerobic medium (GAM) broth (Nissui Pharmaceutical Industrial Co., Tokyo, Japan) for 48–72 h in an anaerobic chamber. The supernatant was then collected by centrifugation at 10000 rpm, 4°C for 50 min and filter-sterilized through a 0.22-μm pore-size membrane filter (Merck Millipore, Darmstadt, Germany). The bacteria examined are listed in Table 1.

**Table 1:** Strains used in this study.

Microorganism	Strain	Source^a^
Methicillin-resistant *Staphylococcus aureus*	HUYM (SCC mec type III)	A
	NU1643 (SCC mec type II; TSST+)	B
	NU1516 (SCC mec type IV; PVL+)	B
*Prevotella intermedia*	PINU499	B

a A, Department of Respiratory Medicine, Faculty of Medicine and Graduate School of Medicine, Hokkaido University, Sapporo, Japan; B, Department of Laboratory Medicine, Nagasaki University Hospital, Nagasaki, Japan.

### Mice

Six-week-old male ddY specific-pathogen-free mice were obtained from SLC Japan Inc., Shizuoka, Japan. All mouse experiments were performed in accordance with the guidelines of the Laboratory Animal Center for Biomedical Research, Hokkaido University School of Medicine. The experimental protocol was approved by the Ethics Committee on Animal Research of our institution.

### Intratracheal Infection Procedure

Intratracheal infection was performed as previously described with minor modifications (Nagaoka et al., 2014a; Yamashita et al., 2019). The MRSA strain was cultured on a sheep blood agar plate (Nissui Pharmaceutical Industrial Co.) for 24 h at 37°C, then scraped and suspended in trypticase soy broth (TSB), and cultured with shaking for 8 h at 37°C and 250 rpm. Bacteria were harvested by centrifugation (3000 rpm, 10 min) and resuspended in normal saline to approximately 10^8^–10^9^ CFU/ml, as determined by measuring optical density with DensiCHEK Plus (bioMérieux Japan, Tokyo, Japan). Mice were anesthetized with ketamine/xylazine, and 0.05 ml of the bacterial suspension was inoculated *via* a 24-gauge catheter inserted in the trachea. In experiments that examined the effect of Pi Sup on MRSA pneumonia, a bacterial suspension of MRSA was mixed with the same amount of Pi Sup or modified GAM broth before inoculating the mice. The final bacterial load of MRSA was approximately 1–2 × 10^9^ CFU/ml (5 × 10^7^–1 × 10^8^ CFU/mouse). The control group was inoculated with equal volumes of GAM broth and normal saline. For the group inoculated with Pi Sup without MRSA, equal volumes of Pi Sup and modified GAM broth were used. Before infection, mice were administered cyclophosphamide intraperitoneally (-day 4: 150 mg/kg; -day 1: 100 mg/kg).

### Bacteriological and Histopathological Examinations

Each group of animals was sacrificed at specific time intervals by cervical dislocation. After exsanguination, the lungs were dissected and removed under aseptic conditions. The lungs used for bacteriological analyses were homogenized with a Precellys 24 high-throughput homogenizer (Bertin Technologies, Rockville, MD, United States). For homogenization, the lungs were transferred into 7-ml Precellys homogenization tubes (CK28; ceramic beads of 2.8 mm; Bertin Technologies) with 2000 μl of sterile saline, and then processed twice at 5500 rpm for 20 s. The homogenized suspension was quantitatively plated onto blood agar plates by serial dilution, followed by incubation at 37°C for 24 h.

### Bronchoalveolar Lavage and Cytokine Enzyme-Linked Immunosorbent Assay

Bronchoalveolar Lavage (BAL) was performed as described previously (Nagaoka et al., 2014a; Yamashita et al., 2019). Recovered fluid fractions were pooled for each animal. Total cell counts were determined using a LUNA-FL automated fluorescence cell counter (Logos Biosystems, Annandale, VA, United States). For differential cell counts, cells were centrifuged at 800 rpm for 10 min onto slides that were then stained with Diff-Quick stain. Counts were performed on 300 cells. Concentrations of macrophage inflammatory protein (MIP)-2 and interleukin (IL)-6 in BAL fluid (BALF) were assayed using mouse cytokine Enzyme-Linked Immunosorbent Assay (ELISA) kits (R and D Systems, Minneapolis, MN, United States) according to the manufacturer’s instructions.

### Effect of Pi Sup on Bacterial Growth of MRSA *in vitro*

Studies on the growth of MRSA were performed in TSB mixed with an equal amount of Pi Sup or modified GAM broth. MRSA was cultured for 3–24 h with shaking at 37°C and 250 rpm from an initial concentration of 2 × 10^5^ CFU/ml. Then, bacterial colony counts were performed by plating serial dilutions on agar plates. The bacterial growth curve of each strain was determined based on results of the quantitative culture.

### Toxin/rnaIII Transcript Levels in MRSA Exposed to Pi Sup *in vitro*

Toxin transcript levels in MRSA strains HUYM, NU1643, and NU1516 grown in TSB were assessed by quantitative reverse transcription PCR, using the primers of gyr, hla, spa, tst, and pvl, which were previously reported (Goerke et al., 2000; Li et al., 2011; Ferreira et al., 2013). The agr quorum sensing system plays a central role in regulating MRSA toxin production (Novick, 2003; Thoendel and Horswill, 2013). Among the various regulatory systems, RNAIII, the small regulatory RNA, acts as an enhancer of several toxins, such as hla, TSST, and PVL; while suppressing other virulent factors including spa (Queck et al., 2008). To confirm the association between toxin and quorum sensing systems, we validated the rnaIII transcript level, using the primers of rnaIII, which were previously reported (Ferreira et al., 2013). Briefly, total RNA was extracted from MRSA using the NucleoSpin RNA kits (Takara Co., Tokyo, Japan) according to the manufacturer’s instructions. Total RNA (1 μg) was reverse-transcribed into cDNA using TaqMan reverse transcription reagents and reverse transcription reaction mix (Thermo Fisher Scientific, Waltham, MA, United States) on an ABI 2720 thermal cycler (Thermo Fisher Scientific, Waltham, MA, United States). The resulting cDNA was used as a template for the StepOnePlus Real-Time PCR System (Thermo Fisher Scientific, Waltham, MA, United States), together with the SYBR Green PCR Master Mix (Thermo Fisher Scientific, Waltham, MA, United States). To quantify the expression of target genes, the following PCR primers were used: gyr (Goerke et al., 2000), TTATGGTGCTGGGCAAATACA, and CACCATGTAAACCACCAGATA; hla (Ferreira et al., 2013), TTTGTCATTTCTTCTTTTTCCCA, and AAGCATCCAAACAACAAACAAAT; spa (Ferreira et al., 2013), TGGTTTGCTGGTTGCTTCTTA, and GCAAAAGCAAACGGCACTAC; tst (Li et al., 2011), TCGCTACAGATTTTACCCCTGT, and CGTTTGTAGATGCTTTTGCAGT; pvl (Li et al., 2011), TGTATCTCCTGAGCCTTTTTCA, and CAGACAATGAATTACCCCCATT; and rnaIII (Ferreira et al., 2013), AATTTGTTCACTGTGTCGATAAT, and TGGAAAATAGTTGATGAGTTGTT. Data are presented as ratios relative to gyrB.

### Lung Toxin/rnaIII Transcript Levels in Mice Infected With MRSA and Treated or Not With Pi Sup *in vivo*

Lung toxin/rnaIII transcript levels in mice were examined 16 h after MRSA inoculation, with or without Pi Sup. Each group of animals was sacrificed at specific time intervals; the lungs were removed and placed into a 7-ml Precellys homogenization tube (CK28) with 2 ml of RNA later, and then processed twice at 5500 rpm for 20 s. The homogenized suspension was transferred into 2-ml Precellys homogenization tubes (CK01; ceramic beads of 0.1 mm; Bertin Technologies), and again processed twice at 6000 rpm for 30 s. Using 100 μl of the homogenized suspension, total RNA was extracted using NucleoSpin RNA kits according to the manufacturer’s instructions. After RNA extraction, cDNA synthesis was carried out, and mRNA transcript levels of hla, spa, tst, pvl, rnaIII, and gyrB were determined by quantitative real-time PCR as described above.

### Effect of Pi Sup on the Bactericidal Activity of Human Polymorphonuclear Leukocytes

All studies with human blood and polymorphonuclear leukocytes (PMNs) were performed in accordance with a protocol approved by the Ethics Committee of our institution. Human PMNs isolation and bactericidal activity assays using MRSA were performed as previously described with minor modifications (Kobayashi et al., 2016). Briefly, PMNs were separated from heparinized whole-blood cells of healthy donors using a density gradient method based on Percoll (GE Healthcare, Chicago, IL, United States) and Ficoll-Paque PLUS (GE Healthcare). PMNs were suspended at 2.5 × 10^6^ cells/ml in phosphate-buffered saline (PBS). MRSA was opsonized in fresh 5% normal human serum for 30 min at 37°C, washed in PBS, and resuspended in RPMI 1640 medium (Sigma-Aldrich, St. Louis, MO, United States) at 5 × 10^4^ CFU/ml. PMNs (5 × 10^5^ cells/200 μl) or 200 μl of PBS were added to 24-well tissue culture plates and combined with 5 × 10^3^ CFU/100 μl MRSA and Pi Sup or 100 μl modified GAM broth. Assay plates were centrifuged at 540 × *g* for 8 min at 4°C. Samples were incubated at 37°C with 5% atmospheric CO2 for 2 h, after which 600 μl of cold distilled water was added to each well. Bacterial colony counts were performed by plating serial dilutions on agar plates.

### Statistical Analysis

All data are expressed as the mean and SEM. Differences between two groups were evaluated using the Mann-Whitney U test; differences among multiple groups (more than two) were evaluated using Steel’s multiple-comparison test. Survival analysis was performed using the log-rank test, and survival rates were calculated using the Kaplan-Meier method. Values of *p* < 0.05 were considered statistically significant.

## Results

### The Supernatant of *P. intermedia* Induces Severe MRSA Pneumonia in a Murine Model

**Figure 1:**
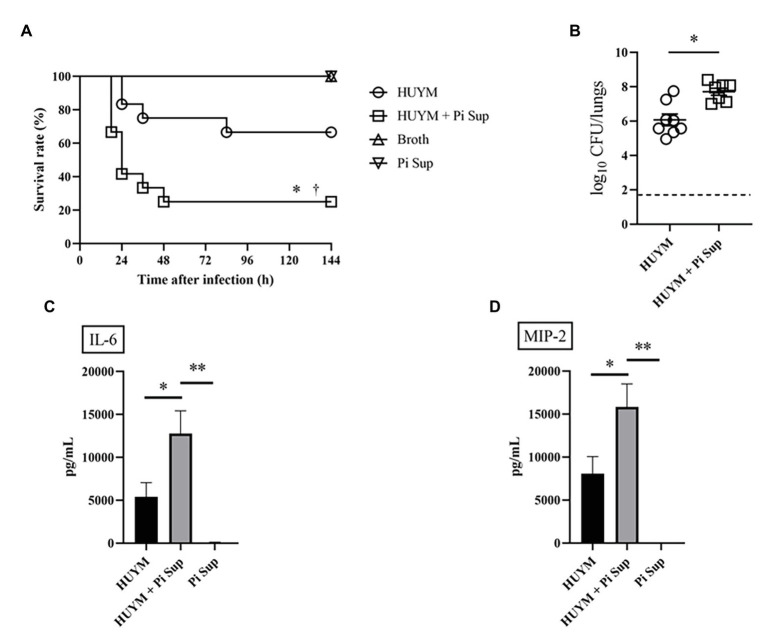
Survival rates **(A)** and bacterial loads **(B)** of mice infected with the HUYM methicillin-resistant *Staphylococcus aureus* (MRSA) strain with/without supernatant of *Prevotella intermedia* (Pi Sup). O, HUYM-infected mice without Pi Sup; ◽, HUYM-infected mice with Pi Sup; △, broth-inoculated mice; and ▽, Pi Sup-inoculated mice. In **(A)** ^*^p < 0.05, HUYM-infected mice with Pi Sup vs. control group; ^†^*p* < 0.05, HUYM-infected mice with Pi Sup vs. HUYM-infected mice without Pi Sup; and *n* = 6–12 mice/group. In **(B)** ^*^p < 0.05; bars represent mean bacterial counts, the broken horizontal line represents the lower limit of detection (1.7 log10 CFU/ml of lung specimen). Similar results were obtained from two independent experiments. Changes in inflammatory cytokine levels in BALF: **(C)** Interleukin (IL)-6 and **(D)** MIP-2. ^*^p < 0.05 and ^**^p < 0.005. Data represent the mean ± SEM of 6–8 mice. Similar results were obtained from two independent experiments.

**Figure 2:**
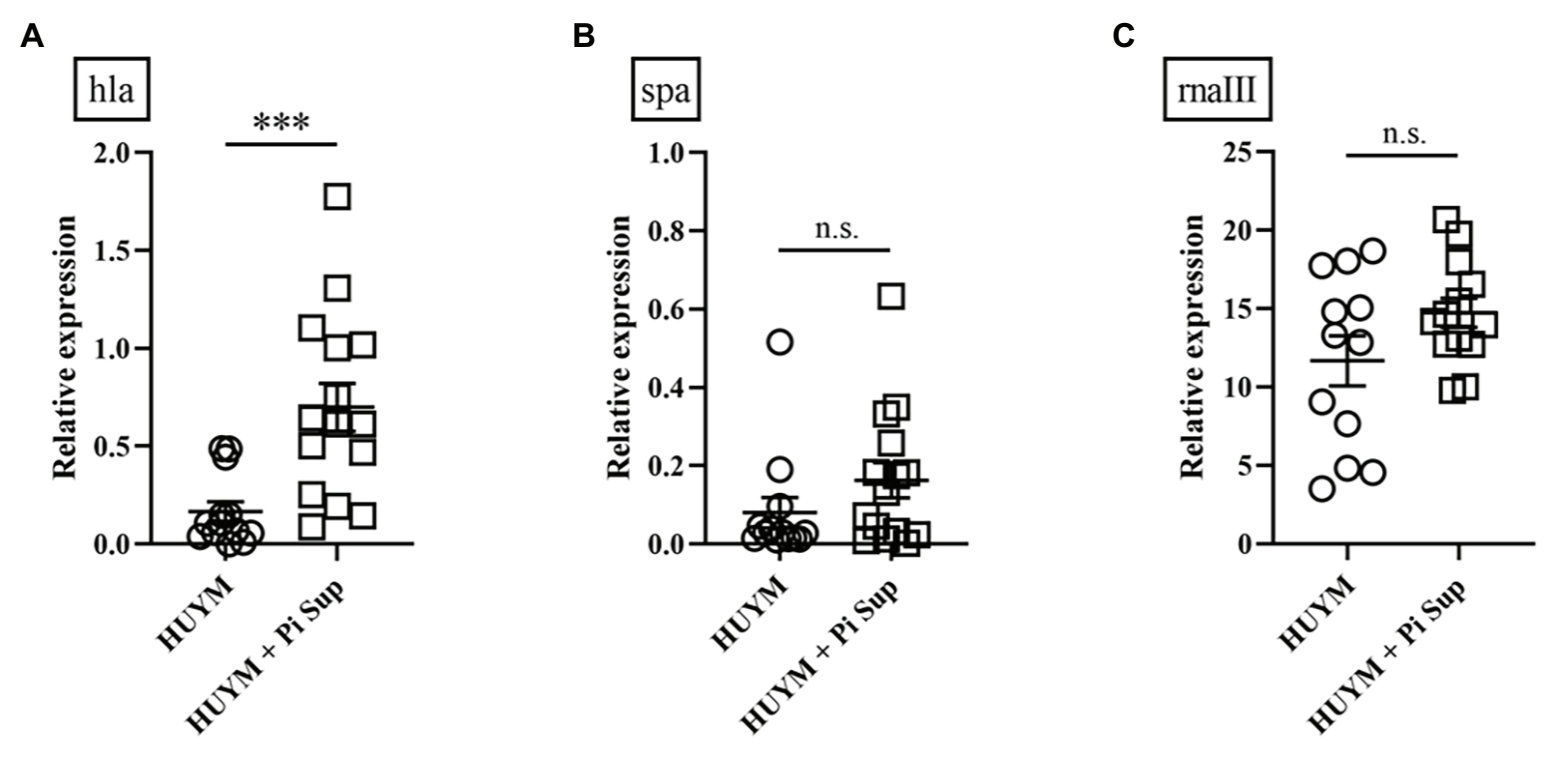
Transcript levels of **(A)** hla, **(B)** spa, and **(C)** rnaIII measured 16 h after inoculation in mice infected with the HUYM MRSA strain with/without Pi Sup. ^***^p < 0.001; n.s., not significant; Bars represent the mean relative messenger RNA (mRNA) expression. Data are representative of 6–8 mice from two independent experiments.

First, we examined the effect of Pi Sup on an MRSA pneumonia murine model. Leucocytes, neutrophil, and lymphpocyte concentrations in blood of BALB/c mice after the administration of cyclophosphamide are listed in Table 2. As shown in Figure 1A, survival rates were significantly lower in MRSA-infected mice treated with Pi Sup than in those without Pi Sup (*p* < 0.05). Conversely, mean bacterial counts in the lungs increased significantly in MRSA-infected mice treated with Pi Sup compared with those without Pi Sup (*p* < 0.05, Figure 1B), and a similar trend was observed for the concentrations of IL-6 (*p* < 0.05, Figure 1C) and MIP-2 (*p* < 0.005, Figure 1D). The total cell and neutrophil counts (Table 3) were not significantly different between MRSA pneumonia with/without Pi Sup. Analysis of toxin transcript levels revealed a significantly higher hla/gyrB ratio in MRSA-infected mice treated with Pi Sup compared with those not subjected to Pi Sup (*p* < 0.001, Figure 2A), whereas no significant difference was observed for the spa/gyrB (Figure 2B) or rnaIII/gyrB ratios (Figure 2C).

**Table 2:** Leukocyte, neutrophil, and lymphocyte concentrations in the blood of ddY mice following administration of cyclophosphamide.

	Cell type (no. cells/mm^3^)		
	Leukocytes	Neutrophils	Lymphocytes
Control group	4,080 (3,400, 6,120)	559 (425, 936)	3,419 (2,730, 4,976)
Cyclophosphamide group	567 (397, 1,360)	5 (0, 12)	509 (350, 1,223)

Cyclophosphamide group was administered cyclophosphamide intraperitoneally (-day 4: 150 mg/kg; -day 1: 100 mg/kg), while control group was administered normal saline instead. Data are presented as the median (25th percentile; 75th percentile) and are representative of 10 replicates. Similar results were obtained from two independent experiments.

**Table 3:** Inflammatory cell density of bronchoalveolar lavage fluid from mice infected with MRSA with or without Pi Sup.

	Cell density × 10^4^ cells/ml, mean ± SEM (*n* = 6–8)			
Cell type	MRSA	MRSA + Pi Sup	Pi Sup	Broth
Overall	59.2 ± 8.7	46.1 ± 12.9	40.5 ± 6.4	63.4 ± 12.0
Neutrophils	20.7 ± 5.2^b^	16.7 ± 8.7^b^	2.6 ± 1.4^a^^,^^b^	0.13 ± 0.07
Macrophages	38.4 ± 5.8	29.3 ± 4.8	37.4 ± 5.9	63.0 ± 11.9
Lymphocytes	0.18 ± 0.07	0.08 ± 0.04	0.46 ± 0.19	0.25 ± 0.08

Data are representative of 6–8 replicates. MRSA, methicillin-resistant Staphylococcus aureus; Pi Sup, supernatant of *P. intermedia*.

a *p* < 0.05 vs. MRSA-infected mice group.

b *p* < 0.05 vs. untreated mice group. Similar results were obtained from two independent experiments.

### Pi Sup Suppresses Human PMN Phagocytosis, MRSA Bacterial Growth, and Virulence Factor Expression *in vitro*

**Figure 3:**
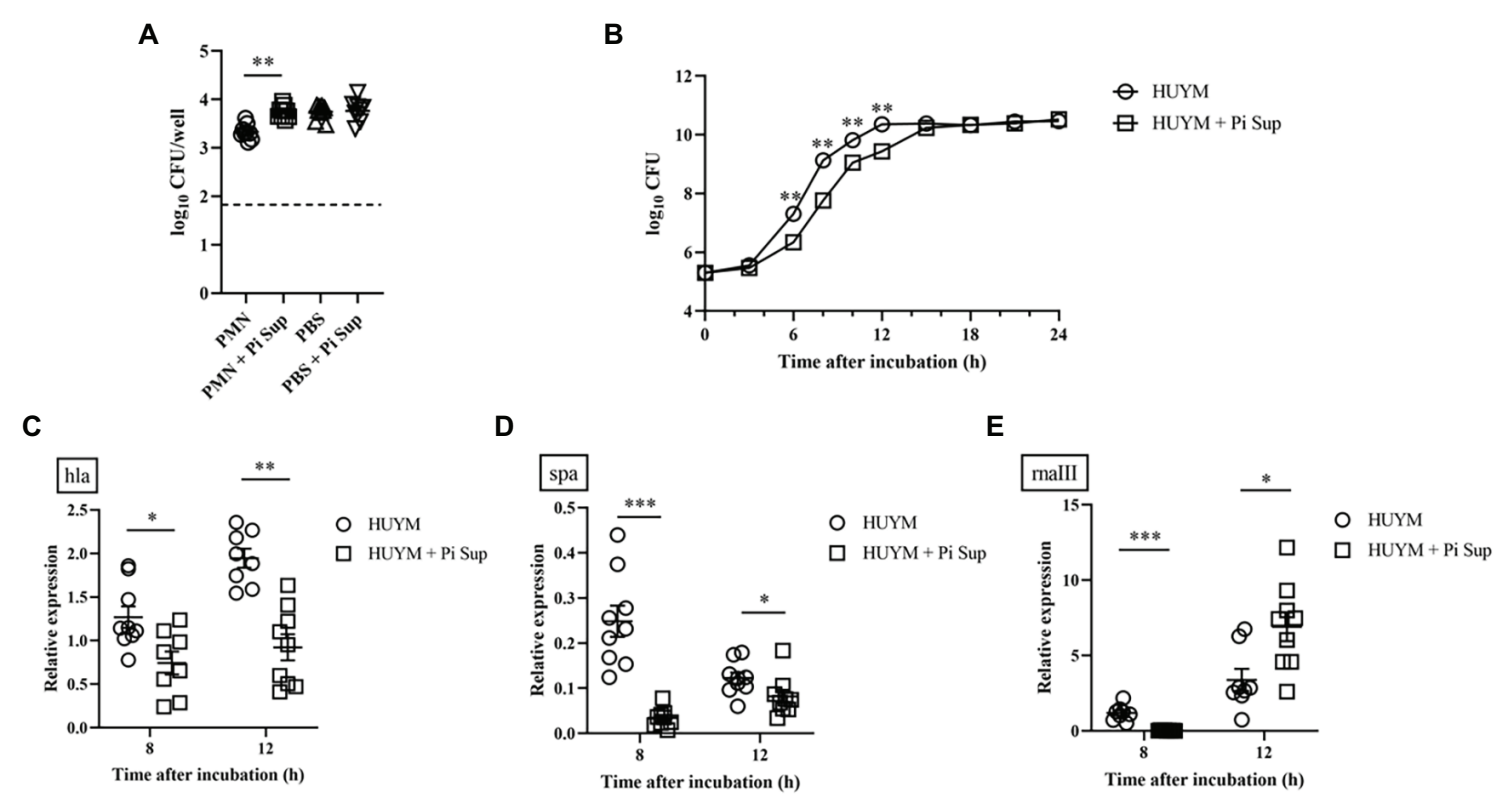
**(A)** Effect of Pi Sup on phagocytosis of human PMNs against the HUYM MRSA strain *in vitro*. Bars represent mean bacterial counts, the broken horizontal line represents the lower limit of detection (1.7 log10 CFU/well). Data are representative of three replicates from three independent experiments. **(B)** Effect of Pi Sup on HUYM growth *in vitro*. O, HUYM without Pi Sup; ◽, HUYM with Pi Sup. Data represent the mean ± SEM of six replicates. Similar results were obtained from two independent experiments. Effect of Pi Sup on **(C)** hla, **(D)** spa, and **(E)** rnaIII mRNA expression *in vitro*. Bars represent the mean relative mRNA expression. Data are representative of three replicates from three independent experiments. ^*^p < 0.05; ^**^p < 0.005; and ^***^p < 0.001.

To examine in greater detail the mechanism by which *P. intermedia* affected MRSA, the effect of Pi Sup on PMN phagocytosis against MRSA was validated. As shown in Figure 3A, bacterial counts were significantly higher in PMNs incubated with Pi Sup than in those without Pi Sup (*p* < 0.005), indicating that Pi Sup suppressed PMNs phagocytosis against MRSA. Next, we examined the effect of Pi Sup on MRSA growth and toxin expression *in vitro*. As shown in Figure 3B, bacterial growth was significantly suppressed in MRSA incubated with Pi Sup at early log to stationary phase (6–12 h; *p* < 0.005), and a similar pattern was seen for hla (Figure 3C) and spa (Figure 3D) transcript levels. In contrast, rnaIII transcript levels were higher in stationary phase of MRSA treated with Pi Sup than in untreated samples (*p* < 0.05, Figure 3E).

### Pi Sup Does Not Affect TSST/PVL-Producing MRSA Strains *in vivo*

**Figure 4:**
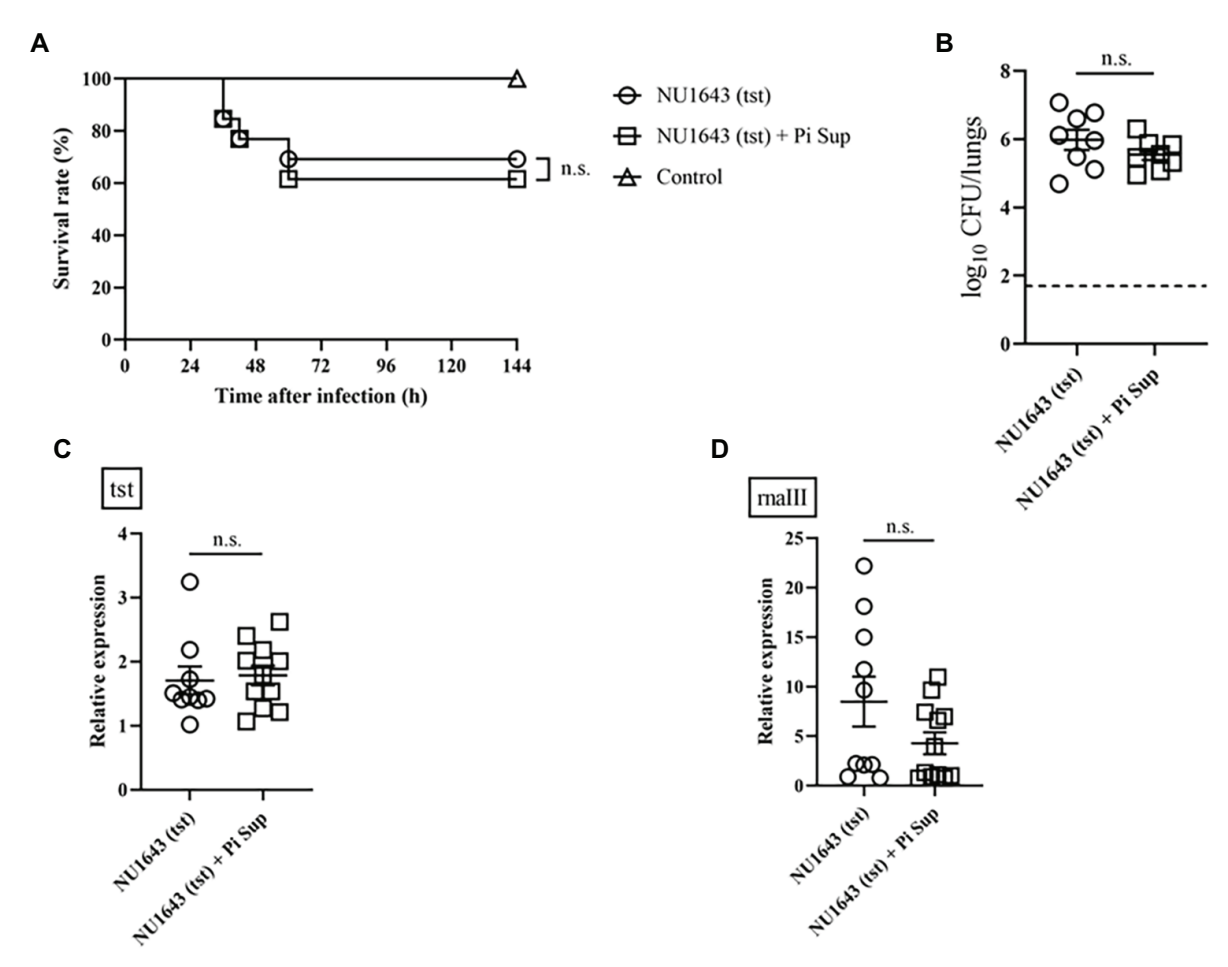
**(A)** Survival rates and **(B)** bacterial loads of mice infected with the NU1643 TSST-producing MRSA strain with/without Pi Sup. O, NU1643-infected mice without Pi Sup; ◽, NU1643-infected mice with Pi Sup; and △, broth-inoculated mice. Bars represent mean bacterial counts, *n* = 6–12 mice/group. The broken horizontal line represents the lower limit of detection (1.7 log10 CFU/ml of lung specimen). Similar results were obtained from two independent experiments. Transcript levels of **(C)** tst and **(D)** rnaIII measured 16 h after inoculation in mice infected with NU1643 MRSA with/without Pi Sup. Bars represent the mean relative mRNA expression. Data are representative of 5–8 mice from two independent experiments. n.s., not significant.

**Figure 5:**
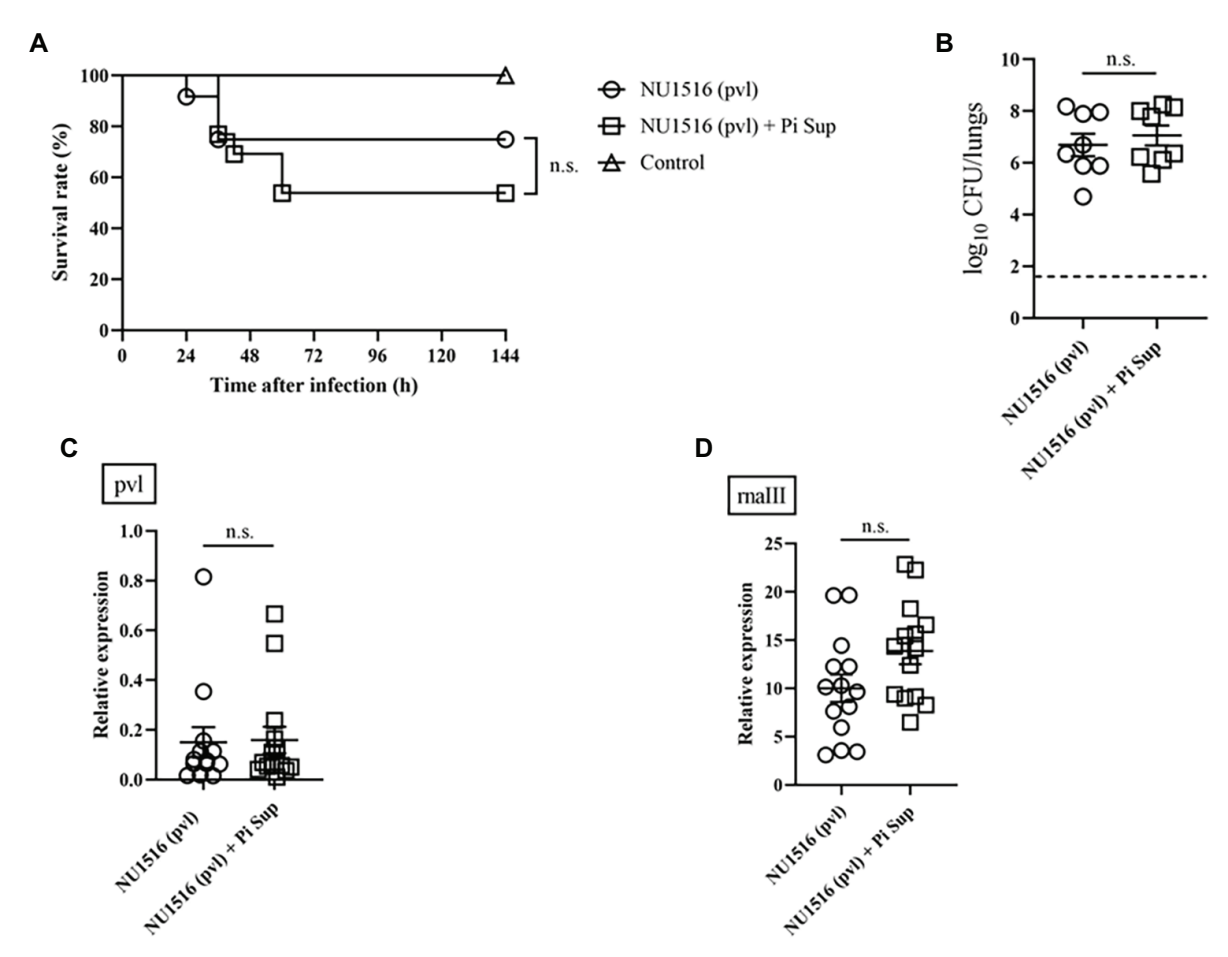
**(A)** Survival rates and **(B)** bacterial loads of mice infected with the NU1516 panton-valentine leucocidin (PVL)-producing MRSA strain with/without Pi Sup. O, NU1516-infected mice without Pi Sup; ◽, NU1516-infected mice with Pi Sup; and △, broth-inoculated mice. Bars represent mean bacterial counts, *n* = 6–12 mice/group. The broken horizontal line represents the lower limit of detection (1.7 log10 CFU/ml of lung specimen). Similar results were obtained from two independent experiments. Transcript levels of **(C)** pvl and **(D)** rnaIII measured 16 h after inoculation in mice infected with NU1516 MRSA with/without Pi Sup. Bars represent the mean relative mRNA expression. Data are representative of 6–8 mice from two independent experiments. n.s., not significant.

To investigate the effect of Pi Sup on MRSA-toxin expression, we used MRSA strains capable of producing more virulent toxins: the TSST-producing NU1643 strain (Figure 4) and the PVL-producing NU1516 strain (Figure 5). Pi Sup exerted no significant effect on survival rates (Figures 4A, 5A), bacterial loads in the lungs (Figures 4B, 5B), tst/pvl expression (Figures 4C, 5C), or rnaIII transcript levels (Figures 4D, 5D) in any of the two strains.

### Strain-Dependent Variation in Lung Proliferation and Toxin/Quorum Sensing Expression *in vivo*

**Figure 6:**
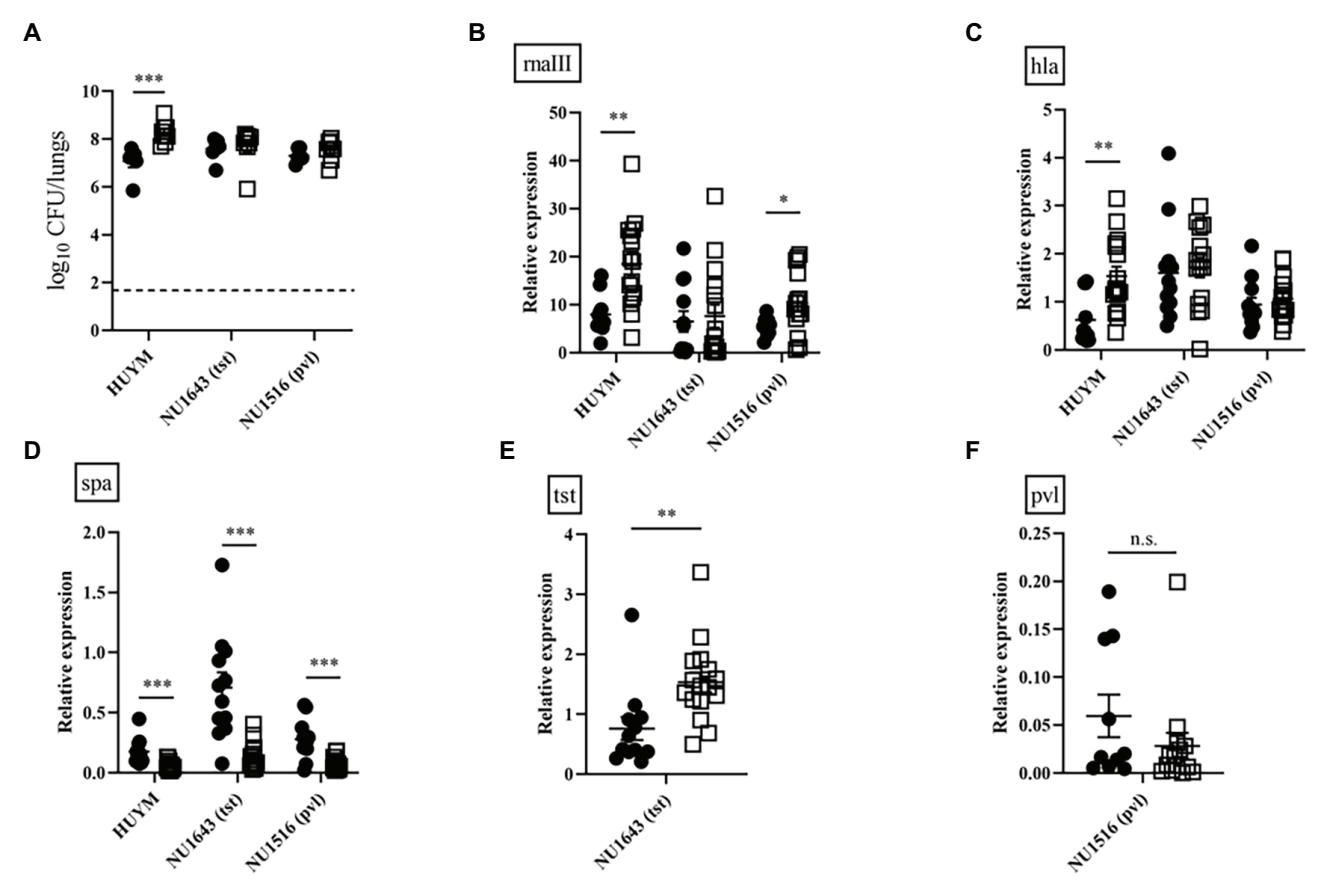
**(A)** Bacterial loads in the lungs of mice infected with the MRSA strains (HUYM, NU1643, and NU1516) at 1 × 10^8^ CFU/mouse for lethal infection. Bacterial counts were validated 4 h (●) and 16 h (◽) after infection. Bars represent mean bacterial counts, *n* = 6–8 mice/group. The broken horizontal line represents the lower limit of detection (1.7 log10 CFU/ml of lung specimen). Similar results were obtained from two independent experiments. Transcript levels of **(B)** rnaIII, **(C)** hla, **(D)** spa, **(E)** tst, and **(F)** pvl measured 4 h (●) and 16 h (◽) after inoculation in mice infected with MRSA strains (HUYM, NU1643, and NU1516). Bars represent the mean relative mRNA expression. Data are representative of 6–8 mice from two independent experiments. ^*^p < 0.05; ^**^p < 0.005; ^***^p < 0.001; and n.s., not significant.

To compare the ability of bacteria to proliferate in the lungs, we examined changes in the number of viable HUYM, NU1643, and NU1516 MRSA in the lungs over time following infection. HUYM was the only strain to achieve a significant increase in bacterial loads between 4 and 16 h after infection (*p* < 0.005, Figure 6A). A significant time-dependent increase in rnaIII (Figure 6B) and hla (Figure 6C) expression was observed in mice infected with the HUYM strain; while spa expression decreased in mice infected with all strains (Figure 6D). A partial increase in rnaIII expression was observed for NU1516 (Figure 6B). NU1643 showed a partial increase in tst expression (Figure 6E), whereas no change was observed regarding pvl expression in NU1516 (Figure 6F). Time-dependent changes in rnaIII, hla, spa, and tst/pvl mRNA expression of each strains *in vitro* were also examined (Supplementary Figures S1–S3). From late log phase (8 h after incubation) until stationary phase (12 h after incubation), all the toxin expression was significantly upregulated, except spa in HUYM strain.

## Discussion

This is the first study to demonstrate that the products of *P. intermedia* can induce severe pneumonia by a hospital-acquired (HA)-MRSA strain. A part of possible mechanisms explaining this outcome is the suppressive effect of Pi Sup on phagocytosis by PMNs.

*In vivo* experiments using the HUYM strain revealed that MRSA-infected mice exhibited significantly lower survival rates, higher MRSA bacterial loads in the lungs, and inflammatory cytokines in BALF when treated with Pi Sup than when not treated. These findings indicate that Pi Sup exerts a pathogenic effect on MRSA pneumonia by enhancing initial MRSA proliferation in the lungs. Unexpectedly, Pi Sup suppressed MRSA growth *in vitro* until stationary phase, as well as expression of both hla and spa virulence factors. However, further experiments revealed that Pi Sup had a suppressive effect on human PMN-mediated phagocytosis against MRSA. Given that exposure to Pi Sup was only temporal, suppression of initial neutrophil phagocytosis could represent the part of effects of Pi Sup on MRSA pneumonia, although continuous exposure to Pi Sup was inhibitive for MRSA growth or toxin expression. In our study, it should be noted that we used cyclophosphamide to establish lethal MRSA-infected model with bacterial proliferation in lungs, which was suitable for investigating the additive pathogenic effect of Pi Sup on MRSA pneumonia. The neutropenic condition generated by exposure to cyclophosphamide (Table 2) affected the initial immune response against MRSA, as indicated by an inadequate increase in inflammatory cells in BALF (Table 3). PMNs play a key role in the initial innate immune response against MRSA pneumonia *via* several pathways, such as bacterial clearance and pulmonary cytokine response (Robertson et al., 2008; Jacqueline et al., 2014). Since complete elimination of neutrophil-activity was not achieved in our cyclophosphamide-treated mice (Tables 2 and 3), enhanced MRSA virulence in the presence of Pi Sup *in vivo* is considered partly due to additional suppression of initial PMN activity. In our previous study (Nagaoka et al., 2017), Pi Sup itself induced inflammation in lower airway tract with upregulation of platelet activating factor receptor, dominant pneumococcal adhesive and invasive factor, which caused severe pneumococcal pneumonia. Similarly, we suggest that the pathogenic effects of Pi Sup on MRSA pneumonia might also have the other pathways *via* the inflammation such as exacerbation of vessel permeability or upregulation of bacterial adhesion factor. Since those factors relating with severe MRSA pneumonia had been still unestablished, further investigation would be necessary in future.

In our study, Pi Sup significantly enhanced hla mRNA expression in mice infected with the HUYM strain, whereas such effect was not significant for spa or rnaIII mRNA expression. Further experiments revealed that Pi Sup did not significantly enhance toxin expression in mice infected with PVL‐ or TSST-producing strains (Figures 4, 5). To investigate these discrepancies, we focused on the lung proliferation ability of each clinical strain, and examined the interaction between quorum sensing and toxin expression. As shown in Figure 6A, the HUYM strain exhibited the strongest lung proliferation *in vivo*, which resulted in a significant time-dependent increase in bacterial loads. RnaIII and hla expression in HUYM-infected mice was also enhanced in a time-dependent manner, while spa expression decreased, indicating toxin regulation by the quorum sensing system. In contrast, time-depending bacterial proliferation was not significant in mice infected by the other clinical strains, and association between tst/pvl and rnaIII expression was generally unclear (Figure 6). *In vitro* time-dependent up-regulation of toxins (hla, tst, and pvl) was confirmed in all clinical strains and was likely associated with rnaIII expression (Supplementary Figures S1–S3). Although spa expression varied among the strains, the other toxins were likely regulated by quorum sensing as previously reported (Queck et al., 2008). Accordingly, we believe that enhanced hla expression in MRSA-infected mice treated with Pi Sup might be induced by activation of the quorum sensing system and this can be achieved only with the HUYM strain.

Quorum sensing of MRSA has attracted renewed attention in recent years as a novel treatment target, and several synthetic, peptide, and natural quorum sensing quenchers have been assessed (Salam and Quave, 2018). However, only a few studies have progressed to *in vivo* MRSA pneumonia models (Waters et al., 2017; Zhou et al., 2018), meaning that a detailed understanding of the role of the agr quorum sensing system in the pathology of MRSA pneumonia remains to be determined. Here, we could demonstrate that Pi Sup promoted MRSA proliferation, resulting in upregulation of toxin expression *via* quorum sensing, which may shed some light on the role of the quorum sensing system in MRSA pneumonia. The observed discrepancies affecting quorum sensing and toxin expression in MRSA-infected mice, might result from the strains’ own varied proliferation in the lungs. Such strain specificity would advocate caution regarding the development of a treatment strategy targeting quorum sensing. Interestingly, the association between quorum sensing and toxin expression in MRSA pneumonia was particularly evident with hla, which is substantially less virulent than PVL or TSST. Improved clinical diagnostic approaches for MRSA pneumonia include novel diagnostic tests, such as an ELISA and an immunochromatographic test targeting PVL (Badiou et al., 2010). Considering the results of our study, a similar diagnostic test targeting hla might be helpful for detecting HA-MRSA pneumonia.

In this study, we used the product of *P. intermedia* instead of the bacterium itself. As described in our previous study (Nagaoka et al., 2014a), *P. intermedia* itself is less virulent under aerobic conditions in the lungs of murine, which had been washed out immediately from lower airway tract after inoculum. Thereby, we could not establish any co-infection model with *P. intermedia* and the other bacterium, including *Streptococcus pneumoniae* and MRSA. Consistently, MRSA pneumonia co-infected with *P. intermedia* occurs only rarely in clinical situation, although the presence of *P. intermedia* in the lower airway tract or saliva is frequently detected by genetic tests (Salminen et al., 2015; Nagaoka et al., 2017). Instead, the product of *P. intermedia* may affect pathogenicity of MRSA pneumonia. As a possible pathway, *P. intermedia* may enhance MRSA pneumonia *via* saliva aspiration, as saliva contains periodontal disease-associated enzymes, cytokines, or other biologically active molecules (Azarpazhooh and Leake, 2006; Kim et al., 2007). Given that aspiration of oral contents occurs frequently in patients under nosocomial settings, the results of our study suggest that the presence of *P. intermedia* in the oral cavity may be a risk factor for severe HA-MRSA pneumonia. Since microbial metabolite is attracting attention as pathogenic factor in several diseases (Ohkusa et al., 2003; Imai et al., 2012), further study is necessary, which determine pathogenic components in Pi Sup.

The present study has several limitations. First, we used a neutropenic mouse model, which is not the most appropriate when exactly examining the effect of Pi Sup on PMNs *in vivo*. Second, we could not validate the amount of toxin by western blot analysis due to its limited abundance even *in vitro*. Sandwich ELISA may be preferable for precise toxin validation in future investigations. Third, we could not adequately evaluate the strain-dependent discrepancies in MRSA pneumonia with Pi Sup, because we only used three MRSA strains for investigation. There is possibility that the other MRSA strains producing TSST or PVL might present similar bacterial proliferation in lungs as well as HUYM strain, which accompanies with TSST/PVL upregulation. We consider it important that the role of toxins in MRSA pneumonia should be further examined. Here, pretreatment with cyclophosphamide was necessary to induce severe MRSA infection of the lower airway tract, and validation of pleural toxin expression by real-time PCR enabled us to demonstrate the partial interaction between *in vivo* MRSA toxin expression and the quorum sensing system.

In conclusion, our results provide novel evidence that the product of *P. intermedia* exerts a pathogenic effect on MRSA pneumonia, in particular with a strain exhibiting strong proliferation in the lower airway tract. Moreover, our results indicate that *P. intermedia* affects MRSA toxin expression *via* quorum sensing in a strain-dependent fashion, which might be important for understanding the pathogenesis of severe MRSA pneumonia.

## Data Availability Statement

All datasets presented in this study are included in the article/Supplementary Material.

## Ethics Statement

The studies involving human participants were reviewed and approved by Ethics Committee of Hokkaido University School of Medicine. The patients/participants provided their written informed consent to participate in this study. The animal study was reviewed and approved by Laboratory Animal Center for Biomedical Research, Hokkaido University School of Medicine.

## Author Contributions

YY and KN designed and interpreted the experiments and prepared the manuscript. YY and KN, with assistance of HK, TF, and KH, performed the majority of the experiments. NK, YM, and KY provided the confirmed MRSA strains. MS, NK, YM, KY, and SK contributed discussions throughout the work. All authors contributed to the article and approved the submitted version.

### Conflict of Interest

The authors declare that the research was conducted in the absence of any commercial or financial relationships that could be construed as a potential conflict of interest.

## Supplementary Material

Supplementary Figure 1 Time-dependent changes in (A) rnaIII, (B) hla, and (C) spa mRNA expression of HUYM MRSA in vitro. Bars represent the mean relative mRNA expression. Data are representative of three replicates from three independent experiments. ^*^*p* < 0.05; ^**^*p* < 0.005.

Supplementary Figure 2 Time-dependent changes in (A) rnaIII, (B) tst, (C) hla, and (D) spa mRNA expression of the NU1643 TSST-producing MRSA strain in vitro. Bars represent the mean relative mRNA expression. Data are representative of six replicates. Similar results were obtained from two independent experiments. ^*^*p* < 0.05; ^**^*p* < 0.005.

Supplementary Figure 3 Time-dependent changes in (A) rnaIII, (B) pvl, (C) hla, and (D) spa mRNA expression of the NU1516 PVL-producing MRSA strain in vitro. Bars represent the mean relative mRNA expression. Data are representative of six replicates. Similar results were obtained from two independent experiments. ^**^*p* < 0.005.
