# Supplementary figures and images for: Pathogenic Effect of Prevotella intermedia on a Mouse Pneumonia Model Due to Methicillin-Resistant Staphylococcus aureus With Up-Regulated α-Hemolysin Expression
Source: Front Microbiol. 2020 Oct 7;11:587235. doi: 10.3389/fmicb.2020.587235 (PMC7575765; doi:10.3389/fmicb.2020.587235)

Figure S1

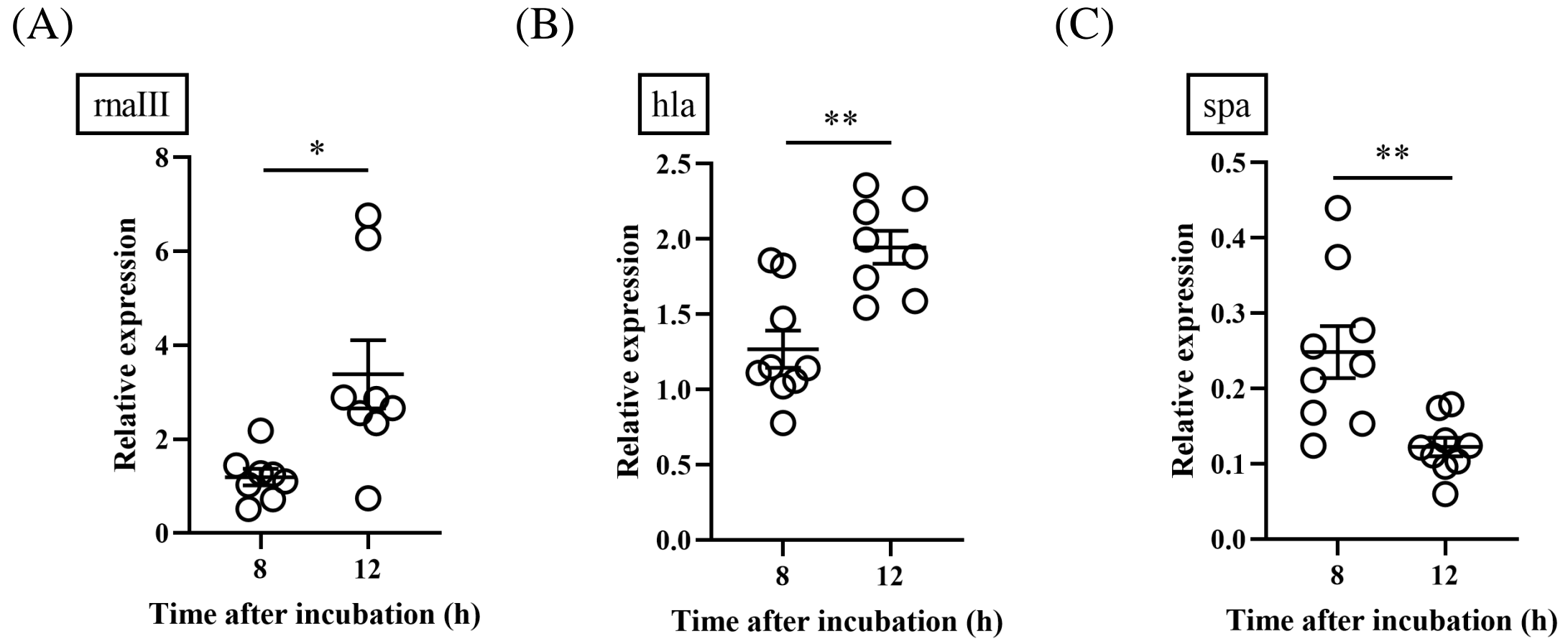

Figure S2

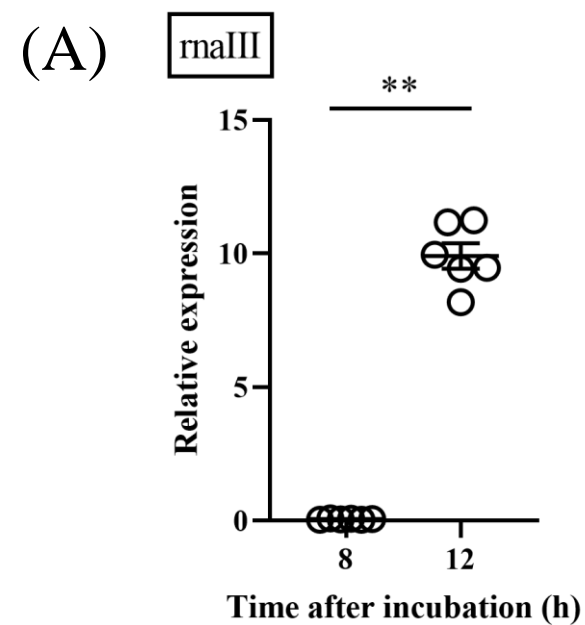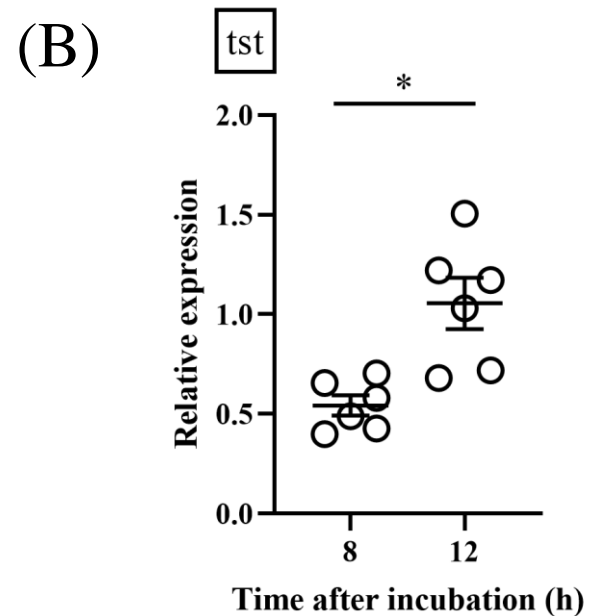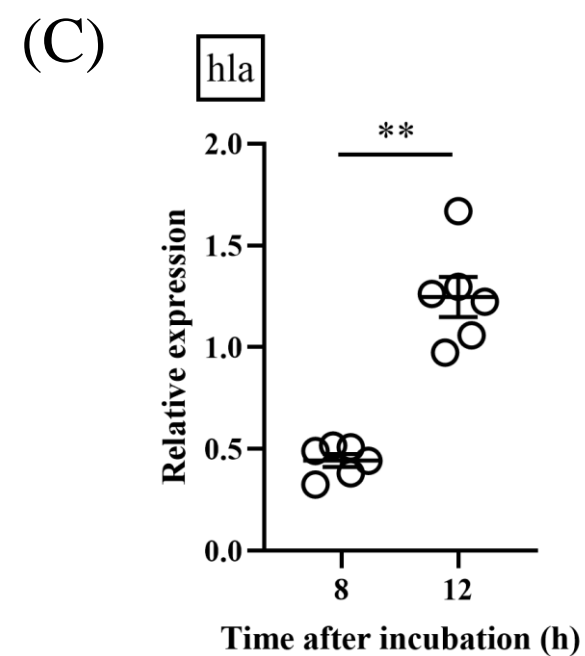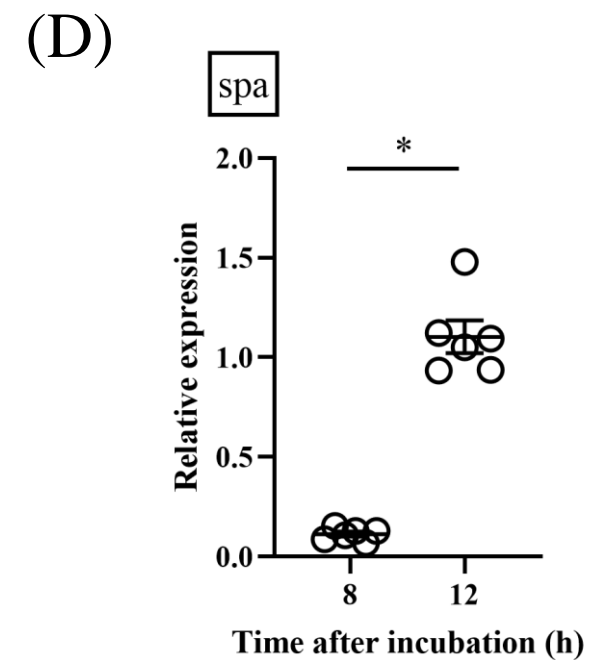

## Figure S3

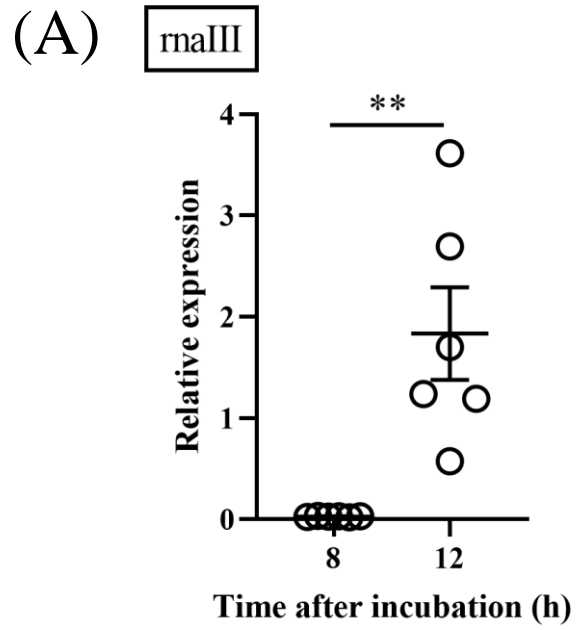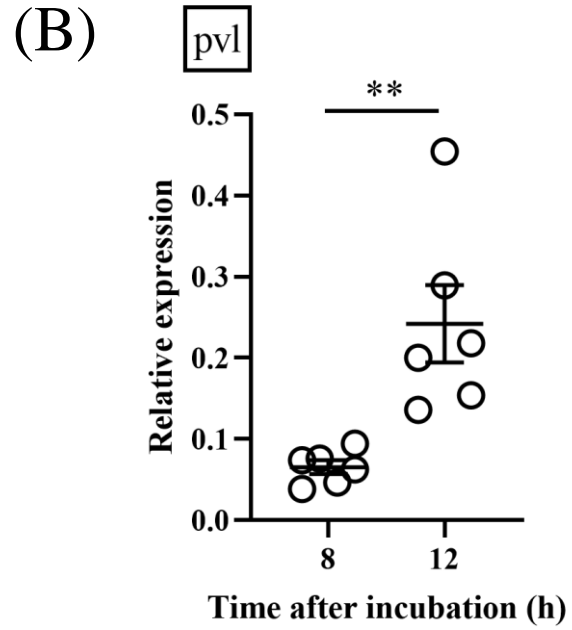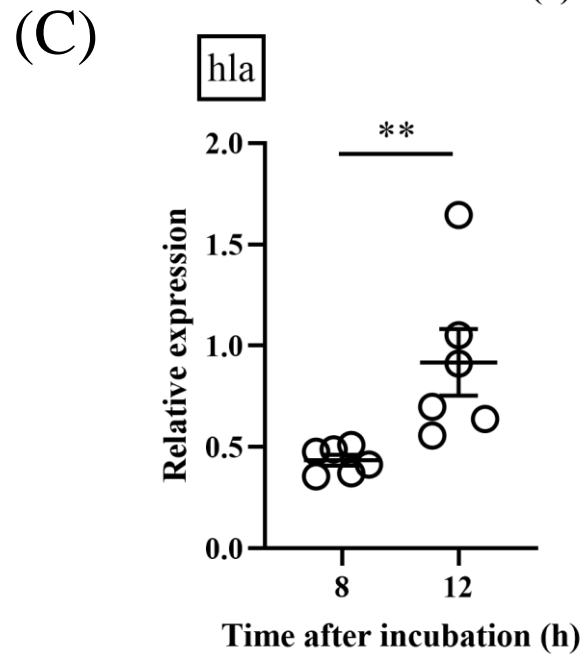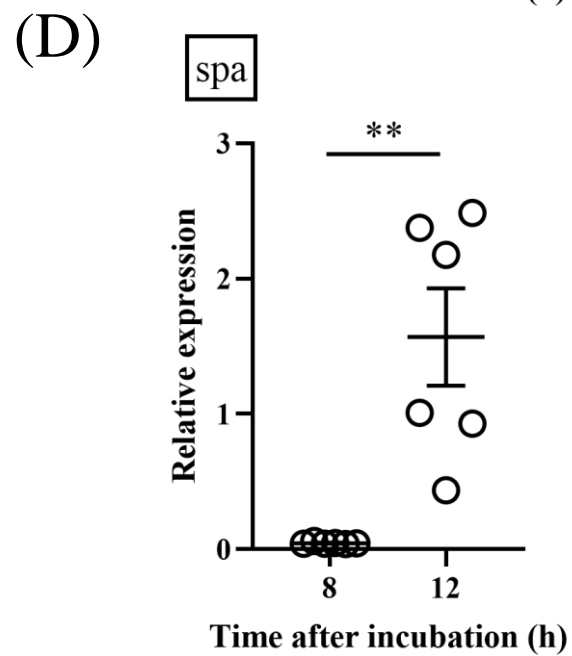

Supplement: Supplementary Figure 1 — Time-dependent changes in (A) rnaIII, (B) hla, and (C) spa mRNA expression of HUYM MRSA in vitro. Bars represent the mean relative mRNA expression. Data are representative of three replicates from three independent experiments. *p < 0.05; **p < 0.005. [file Data_Sheet_1.pdf]
